# QSPR modeling of some COVID-19 drugs using neighborhood eccentricity-based topological indices: A comparative analysis

**DOI:** 10.1371/journal.pone.0321359

**Published:** 2025-05-20

**Authors:** Yeliz Kara, Yeşim Saǧlam Özkan, Asad Ullah, Yasser Salah Hamed, Melaku Berhe Belay

**Affiliations:** 1 Department of Mathematics, Faculty of Arts and Science, Bursa Uludag University, Bursa, Turkey; 2 Department of Mathematical Sciences, Karakoram International University Gilgit-Baltistan, Gilgit, Pakistan; 3 Department of Mathematics and Statistics, College of Science, Taif University, Taif, Saudi Arabia; 4 Nanotechnology Center of Excellence, Addis Ababa Science and Technology University, Addis Ababa, Ethiopia; 5 Mathematics, Physics and Statistics Division; Addis Ababa Science and Technology University, Addis Ababa, Ethiopia; International Center For Chemical and Biological Sciences, PAKISTAN

## Abstract

COVID-19, which emerged in 2019, is a disease caused by a new coronavirus, severe acute respiratory syndrome coronavirus 2 (SARSCoV-2), and has caused a worldwide epidemic. During and after this outbreak, it has been confirmed once again that finding a drug to prevent and end such diseases as soon as possible is an important issue. However, drug discovery and to determine a molecule’s physical characteristics in a lab takes effort and time and is a costly process. Relevant information about molecules can be obtained by calculating topological indices, which are molecular descriptive numerical values corresponding to the physical properties of the chemical structure of a molecule. In this paper, we consider recently used drugs such as arbidol, chloroquine, hydroxy-chloroquine, lopinavir, remdesivir, ritonavir, thalidomide and theaflavin in treatment of COVID-19. This article examines neighborhood eccentricity-based topological descriptors that are used to analyze the structures of potential drugs against COVID-19. Eccentricity-based topological indices are advancing the field of chem-informatics and helping scientists better understand structure-activity correlations across a wide range of chemical compounds. The purpose is to identify structural components that have a significant impact on physico-chemical properties. In this context, the chemical structure and the corresponding molecular graph of the drugs under consideration are given in order to calculate the neighborhood eccentricity values. QSPR models are studied using linear and cubic regression analysis with topological indices for boiling point, enthalpy of vaporization, flash point, molar refraction, polar surface area, polarizability, molar volume and molecular weight properties of these drugs. Regression analysis is applied to find potential correlation between different drug characteristics such as bio-availability and efficacy. The results show that topological indices and applied regression models are useful in predicting significant characteristics of drugs used for the treatment of COVID-19. Additionally, a comparison of the known values and the calculated values from the regression models discussed is obtained.

## 1 Introduction

The disease known as COVID-19 is caused by a novel strain of coronavirus, SARS-CoV-2, which was first identified in China in late 2019. Since then, it has spread to more than 200 countries and territories, resulting in a global pandemic that has affected millions of people and disrupted various aspects of life. The ongoing global pandemic of COVID-19 has undoubtedly been the most significant public health crisis of the 21st century. It has resulted in over 7 million deaths and over 770 million confirmed cases worldwide, creating a significant challenge to global public health [1]. The pandemic has had a profound impact on the physical well-being of millions of people, but it has also affected their psychological, social and economic aspects of life. The World Health Organisation (WHO) and other health authorities have endeavoured to monitor the situation, provide guidance and coordinate the pandemic response. While the development of specific therapeutics and vaccines represents a key objective, achieving mutations of the virus in the world’s population presents a significant challenge. Although some drugs and therapies can help reduce the severity and duration of the disease, it still represents an important target for drug discovery, as the major proteases of the Coronavirus (SARS-CoV-2) play a critical role during the spread of the disease. Since the beginning of this pandemic, hundreds of remarkable papers have been published to justify the cause of viral spread, applicable preventive measures and future therapeutic approaches. In the current era, scientists are engaged in a concerted effort to develop effective diagnostic and therapeutic strategies for the treatment of COVID-19. The high mortality and infection rates associated with this disease have placed new demands on management levels, society, individuals, and researchers in the fight against the pandemic.

The process of discovering and developing new drug molecules is fraught with challenges, including high costs, lengthy periods of time, and limited success in clinical trials. Furthermore, the necessity for new molecules to be more effective, less side-effective and cheaper than existing drugs represents a significant challenge for the pharmaceutical industry, reducing its overall productivity. Despite these challenges, continued drug researches and developments of this field are vital for the quality of life of individuals, public health and governments worldwide.

New variants of the SARS-CoV-2 virus continue to emerge, and there are concerns that these could be resistant to some of the vaccines currently in use for COVID-19. As a result, there is a pressing need to identify a drug that will prevent and halt the spread of this disease as quickly as possible. However, drug discovery is a complex process that requires considerable effort, time, and financial resources. The application of computer-assisted drug design (CADD) has recently been employed to facilitate the process of identifying promising leads, including those with electronic, drug-like, and pharmacokinetic properties. Graph theory, which was first introduced by Euler in 1736, is one of the most widely studied branches of discrete mathematics, with applications across various fields, including physics, biology, computer science, and chemistry [2]. The field of chemical graph theory is a fusion of mathematical modeling of chemical phenomena with graph theory. Relevant information about molecules can be obtained by calculating topological indices, which are molecular descriptive numerical values corresponding to the physical properties of the chemical structure of a molecule and molecule compounds [3]. The primary objective of studying topological indices is to acquire and modify chemical structure data, thereby establishing a mathematical correlation between structures and physico-chemical properties, bio-activities, and other experimental attributes.

In the field of quantitative analysis of the relationships between structure and property, topological indices prove invaluable in elucidating a multitude of physical and chemical properties. Quantitative structure–property relationship (QSPR) analysis is a highly promising approach to the study of structural characteristics and their correlation with the properties of complex materials [4].

The utilization of topological indices has emerged as a pioneering approach in the intricate field of molecular research, offering a quantitative lens through which to interpret the molecular structure subtleties of chemical molecules. In recent times, there has been a great deal of discussion surrounding the use of QSPR models to study the relationships between physico-chemical properties and some topological indices of drugs used in the treatment of COVID-19 [5–13].

Eccentricity-based topological indices [14–16] which represent a class of molecular descriptors, are frequently employed in both analytical and quantitative structure-activity relationship (QSAR) analysis. The eccentricity values of the constituent atoms in a molecular structure are employed in the calculation of these indices. The eccentricity value is the distance between an atom and its nearest neighbor in a molecule. In 2011 [17], Gutman introduced the concept of eccentricity-based topological indices, which established a framework for future studies in the field. The Wiener index is a fundamental eccentricity-based topological index, defined as the total of all the eccentricity of a molecular graph’s vertex combinations. Due to its strong correlation with specific physico-chemical characteristics and bio-activity profiles, the Wiener index has been employed in the fields of drug design and environmental chemistry [18]. The Zagreb indices represent another significant example of an eccentricity-based indicator. The capacity to forecast boiling temperatures, bioavailability, and other molecular features has been demonstrated with the application of these indices. The advancement of cheminformatics and the enhanced comprehension of structure-activity correlations across a diverse array of chemical compounds has been significantly influenced by research on eccentricity-based topological indices [19–31]. Research on eccentricity-based topological indices has been of great significance in advancing the field of cheminformatics and in helping scientists to better comprehend the structure-activity correlations of a wide range of chemical compounds [32–35].

In order to facilitate the analysis of drugs with respect to their physico-chemical properties, particularly in the context of the novel coronavirus (COVID-19), we have selected eight eccentricity-based topological indices. The drugs arbidol, chloroquine, hydroxy-chloroquine, lopinavir, remdesivir, ritonavir, thalidomide, and theaflavin, which are used in the treatment of COVID-19, are discussed in this article.

We introduce a number of neighborhood eccentricity-based topological indices such as first neighborhood eccentricity index, NE_1_, second neighborhood eccentricity index, NE_2_, forgotten neighborhood eccentricity index, NFE, reformulate neighborhood eccentricity index, NRE, symmetric degree division neighborhood eccentricity index, NSD_deg_E, inverse sum indegree neighborhood eccentricity, NISIE, harmonic neighborhood eccentricity, NHE, augmented neighborhood eccentricity, NAE. There are hundreds of topological indices in the literature, and new ones are being created every day. However, it is difficult to perform calculations using formulae each time. To overcome this difficulty, eccentricity based topological indices have been computed by defining the NEC polynomial based on the neighborhood eccentricity of vertices in a graph *G* in this work. By employing these indices derived from the topological analysis of neighborhood eccentricities, we are able to identify the structural characteristics of the drugs against COVID-19. The selected topological indices, when utilized as quantitative descriptor tools, provide a framework for the examination of the drugs’ molecular environments.

The paper is organized as follows: In Sect 2, we give some basic tools of graph theory and molecular structures of the various COVID-19 drugs. In Sect 3, we calculate the values of topological indices for molecular graph structure of these drugs via NEC-polynomials. In Sect 4, chemical suitability among the physicochemical properties of these drugs and eight topological indices are checked via correlation technique and, make mathematical modeling between them by QSPR analyzing technique. Sect 5 is devoted to comparison and discussions. Finally, we conclude the paper in Sect 6.

## 2 Material methods

Let *G* shows the molecular graph. The vertices of a molecular graph, *V*(*G*), represented by unsaturated hydrocarbon skeletons of the molecule and molecular compounds, correspond to non-hydrogen atoms. *E*(*G*) is a set of bonds is termed as edge sets. The length of the shortest path (the number of edges in it) connecting the vertices *u* and *v* of the graph *G* is equal to the distance *d*(*u*, *v*) between them. Two vertices *u* and *v* are connected by an edge e=uv of the graph *G*, i.e., d(u,v)=1. The eccentricity of the atom *u* in *G* is the maximum distance from *u* to all other vertices in *G*, that is, $\epsilon_{u}=max\left\{d(u,v)|u\in V(G)\right\}$. In [16], the *EC*–polynomial with respect to eccentricity of vertices in a graph *G* which is defined as

$$EC(G,x,y)=\sum_{i\leq j}\eta_{ij}x^{i}y^{j},$$ (1)

where $\eta_{ij}=|{ℰ}_{i,j}|$ and ${ℰ}_{i,j}=\left\{uv\in E(G)|\epsilon_{u}=i,\epsilon_{v}=j\right\}$.

The neighborhood eccentricity of a vertex *u* in a graph *G* is defined as the sum of eccentricity of the vertices adjacent to *u* in *G* and denoted by μ(u) in [36], that is $\mu (u)=\sum_{v\in N(u)}\epsilon_{u}$, where N(u)={u∈V(G)|uv∈E(G)}. The *NE*C-polynomial based on the neighborhood eccentricity of vertices in a graph *G* which is introduced as follows:

$$NEC(G,x,y)=\sum_{i\leq j}m_{ij}x^{i}y^{j},$$ (2)

where $m_{ij}=|E_{i,j}|$ and $E_{i,j}=\left\{uv\in E(G)|\mu (u)=i,\mu (v)=j\right\}$.

The following operations are used to find required results for this article:

$$d_{x}=x\frac{\partial h(x,y)}{\partial x},\;\;\;d_{y}=y\frac{\partial h(x,y)}{\partial y},\;S_{x}=\int_{0}^{x}\frac{h(t,y)}{t}dt,\;\;\;S_{y}=\int_{0}^{y}\frac{h(x,t)}{t}dt,\;\Psi_{k}(h(x,y))=x^{k}h(x,y),\;\;\;J(h(x,y))=h(x,x).$$ (3)

The topological indices considered in this study are summarized in Table 1.

**Table 1:** Topological indices and their mathematical expressions.

Topological indices	Mathematical expressions (μ(u),μ(v))	Derivation from
NE_1_	$\sum_{uv\notin E}(\mu (u)+\mu (v))$	$\left(d_{x}+d_{y})(h(x,y))(1,1\right)$
NE_2_	$\sum_{uv\notin E}(\mu (u)\mu (v))$	$\left(d_{x}d_{y})(h(x,y))(1,1\right)$
NFE	$\sum_{uv\notin E}(\mu (u{)}^{2}+\mu (v{)}^{2})$	$\left(d_{x}^{2}+d_{y}^{2})(h(x,y))(1,1\right)$
NRE	$\sum_{uv\notin E}(\mu (u)\mu (v)(\mu (u)+\mu (v)))$	$\left(d_{x}d_{y}(d_{x}+d_{y}))(h(x,y))(1,1\right)$
NSD_deg_E	$\sum_{uv\notin E}(\frac{\mu (u)}{\mu (v)}+\frac{\mu (v)}{\mu (u)})$	$\left(d_{x}S_{y}+S_{x}d_{y})(h(x,y))(1,1\right)$
NISIE	$\sum_{uv\notin E}(\frac{\mu (u)\mu (v)}{\mu (u)+\mu (v)})$	$\left(S_{x}Jd_{x}d_{y})(h(x,y))(1,1\right)$
NHE	$\sum_{uv\notin E}(\frac{2}{\mu (u)+\mu (v)})$	(2*S*_*x*_*J*)(*h*(*x*,*y*))(1,1)
NAE	$\sum_{uv\notin E}(\frac{\mu (u)\mu (v)}{\mu (u)+\mu (v)-2}{)}^{3}$	$\left(S_{x}^{3}\Psi_{-2}Jd_{x}^{3}d_{y}^{3})(h(x,y))(1,1\right)$

In this part of the study, structural properties and molecular graphs of arbidol, chloroquine, hydroxy-chloroquine, lopinavir, remdesivir, ritonavir, thalidomide, and theaflavin drugs used to alleviate the COVID-19 pandemic are given [37]. The antiviral agent arbidol is currently being investigated as a potential treatment and prophylactic agent for coronavirus disease 2019 (COVID-19) in conjunction with both existing and investigational antiretroviral therapies for human immunodeficiency virus (HIV). Chloroquine, the most widely used drug against malaria, is also used in the treatment of autoimmune disease. Hydroxy-chloroquine is a medication that has been employed in the treatment of malaria since the Second World War. It is also a common prescription for rheumatoid arthritis, chronic discoid lupus erythematosus and systemic lupus erythematosus. Lopinavir was previously under investigation in combination with ritonavir for the treatment of COVID-19. Remdesivir has been demonstrated to function as a non-obligate chain terminator of RdRp (RNA-dependent RNA polymerase) from SARS-CoV-2 and the related SARS-CoV and MERS-CoV, and has been subjected to investigation within multiple clinical trials for the treatment of COVID-19. Remdesivir is a nucleoside analogue drug that impairs the replication of viral RNA. Thalidomide, which is known to cause congenital defects (phocomelia) in the fetus, is employed in the treatment of numerous autoimmune disorders, including psoriasis and systemic lupus erythematosus. Theaflavin is a pharmaceutical agent employed in the treatment of conditions where absorption is impaired, such as oral health, gastric ulcers, and intestinal or colonic disorders [37].

**Fig 1:**
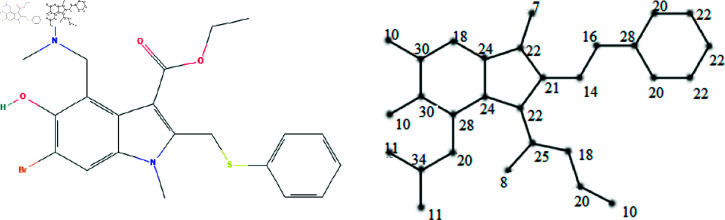
The chemical structure and corresponding molecular graph of arbidol, respectively [37].

**Fig 2:**
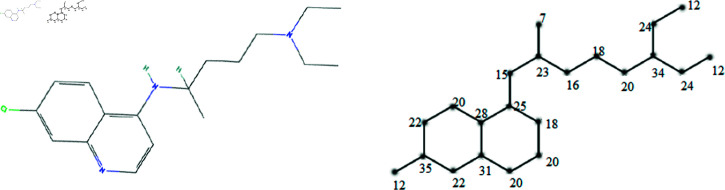
The chemical structure and corresponding molecular graph of chloroquine, respectively [37].

**Fig 3:**
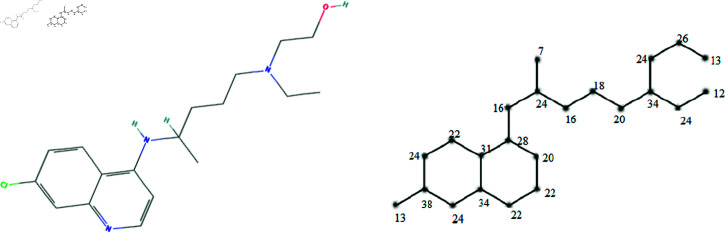
The chemical structure and corresponding molecular graph of hydroxy-chloroquine, respectively [37].

**Fig 4:**
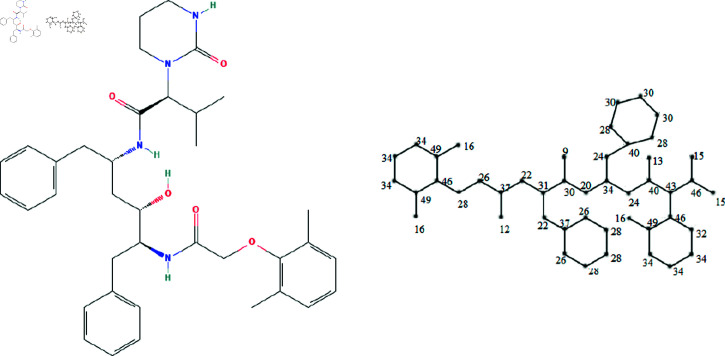
The chemical structure and corresponding molecular graph of lopinavir, respectively [37].

**Fig 5:**
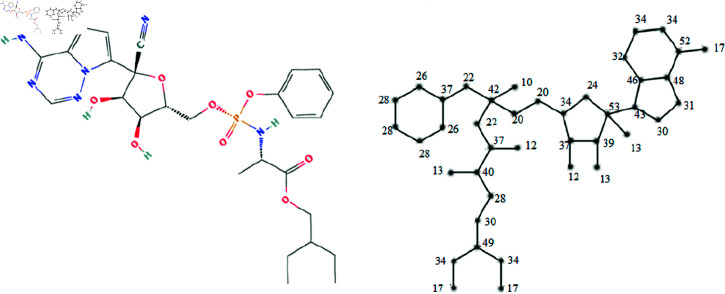
The chemical structure and corresponding molecular graph of remdesivir, respectively [37].

**Fig 6:**
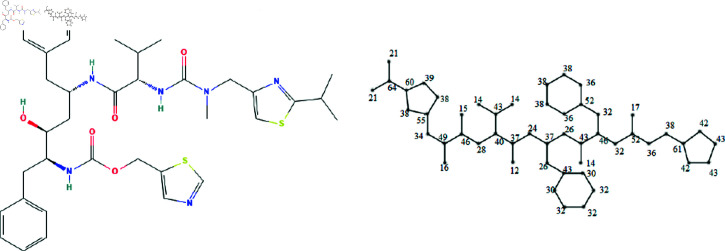
The chemical structure and corresponding molecular graph of ritonavir, respectively [37].

**Fig 7:**
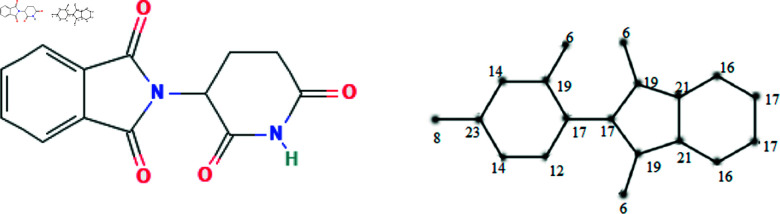
The chemical structure and corresponding molecular graph of thalidomide, respectively [37].

**Fig 8:**
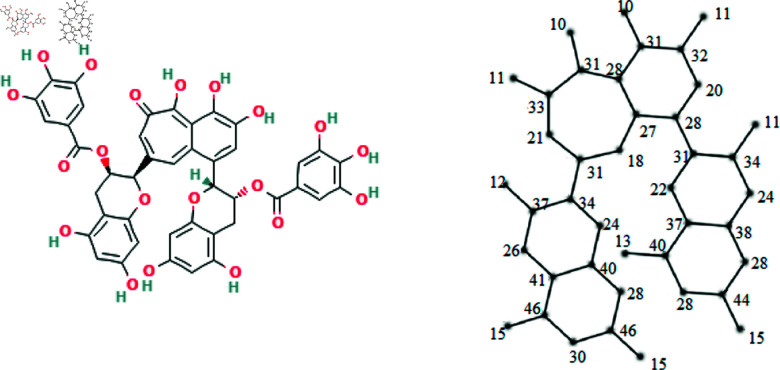
The chemical structure and corresponding molecular graph of theaflavin, respectively [37].

The molecular structures of the eight drugs used for the treatment of COVID-19 have been taken into consideration and presented in Figs 1,2,3,4,5,6,7, and 8. Indeed, they facilitate the study of reactions, the understanding of structure-activity relationships, and the analysis of molecular characteristics in a manner that would otherwise prove challenging. We employee the edge partitioning technique for eccentric-based indices to calculate the values for the aforementioned topological indices. Note that the neighborhood eccentricity of each vertex of each molecular graphs is written in the Figs 1,2,3,4,5,6,7, and 8.

## 3 Computation of topological indices

In this section, the NEC-polynomials of the chemical structures of arbidol, chloroquine, hydroxy-chloroquine, lopinavir, remdesivir, ritonavir, thalidomide, and theaflavin drugs are obtained. Using these polynomials, various topological indices depending on the eccentricity are calculated for these structures.

**Theorem 1.** *Let G_1_ be the graph of arbidol. NEC-polynomial of G_1_ is given as follows:*

$$NEC(G_{1},x,y)=x^{7}y^{22}+x^{8}y^{25}+x^{10}y^{20}+2x^{10}y^{30}+2\;x^{11}y^{34}+x^{14}y^{16}+x^{14}y^{21}\;\;\;+x^{16}y^{28}+x^{18}y^{24}+x^{18}y^{25}+x^{18}y^{20}+x^{18}y^{30}+2\;x^{20}y^{22}+3\;x^{20}y^{28}\;\;\;+x^{20}y^{34}+2\;x^{21}y^{22}+2\;x^{22}y^{22}+2\;x^{22}y^{24}+x^{22}y^{25}+x^{24}y^{24}+x^{24}y^{28}\;\;\;+x^{28}y^{30}+x^{30}y^{30}.$$ (4)

*Proof:* From Fig 1, it is easy to see that |V|=29 and |E|=31. The frequencies are given as |*E*_7,22_| = 1, |*E*_8,25_| = 1, |*E*_10,20_| = 1, |*E*_10,30_| = 2, |*E*_11,34_| = 2, |*E*_14,16_| = 1, |*E*_14,21_| = 1, |*E*_16,28_| = 1, |*E*_18,20_| = 1, |*E*_18,24_| = 1, |*E*_18,25_| = 1, |*E*_18,30_| = 1, |*E*_20,22_| = 2, |*E*_20,28_| = 3, |*E*_20,34_| = 1, |*E*_21,22_| = 2, |*E*_22,22_| = 2, |*E*_22,24_| = 2, |*E*_22,25_| = 1, |*E*_24,24_| = 1, |*E*_24,28_| = 1, |*E*_28,30_| = 1 and |*E*_30,30_| = 1. Then, we have


$$NEC(G_{1},x,y)=\sum_{i\leq j}m_{ij}x^{i}y^{j}\;=x^{7}y^{22}+x^{8}y^{25}+x^{10}y^{20}+2x^{10}y^{30}+2\;x^{11}y^{34}+x^{14}y^{16}+x^{14}y^{21}\;\;\;+x^{16}y^{28}+x^{18}y^{24}+x^{18}y^{25}+x^{18}y^{20}+x^{18}y^{30}+2\;x^{20}y^{22}+3\;x^{20}y^{28}\;\;\;+x^{20}y^{34}+2\;x^{21}y^{22}+2\;x^{22}y^{22}+2\;x^{22}y^{24}+x^{22}y^{25}+x^{24}y^{24}+x^{24}y^{28}\;\;\;+x^{28}y^{30}+x^{30}y^{30}.$$

◻

**Theorem 2.** *Let G_1_ be the graph of arbidol. Using Fig 1, the various topological indices of the G_1_ graph are as follows:*

1. NE_1_(G_1_)=1355
2. NE_2_(G_1_)=14376
3. NFE(G_1_)=32089
4. NRE(G_1_)=664924
5. NSD_deg_E (G_1_)=72.5912
6. NISIE(G_1_)=318.2806
7. NHE(G_1_)=1.4614
8. NAE (G_1_)=43768.2669

*Proof:* The following indices can be calculated with the help of the *NEC*-polynomial obtained in Theorem 1:

$$NE_{1}(G_{1})=((d_{x}+d_{y})NEC(G_{1},x,y))(1,1)\;=(29\;x^{7}y^{22}+33\;x^{8}y^{25}+30\;x^{10}y^{20}+30\;x^{14}y^{16}+35\;x^{14}y^{21}+44\;x^{16}y^{28}\;\;\;+42\;x^{18}y^{24}+43\;x^{18}y^{25}+38\;x^{18}y^{20}+48\;x^{18}y^{30}+54\;x^{20}y^{34}\;\;\;+47\;x^{22}y^{25}+48\;x^{24}y^{24}+52\;x^{24}y^{28}+58\;x^{28}y^{30}+60\;x^{30}y^{30}+80\;x^{10}y^{30}\;\;\;+90\;x^{11}y^{34}+84\;x^{20}y^{22}+144\;x^{20}y^{28}+86\;x^{21}y^{22}\;\;\;+88\;x^{22}y^{22}+92\;x^{22}y^{24})(1,1)=1355.$$ (5)

$$NE_{2}(G_{1})=((d_{x}d_{y})NEC(G_{1},x,y))(1,1)\;=(2\;x^{7}y^{16}(77\;y^{6}+100\;xy^{9}+100\;x^{3}y^{4}+300\;x^{3}y^{14}+374\;x^{4}y^{18}+147\;x^{7}y^{5}\;\;\;+224\;x^{9}y^{12}+225\;x^{11}y^{9}+180\;x^{11}y^{4}+270\;x^{11}y^{14}+440\;x^{13}y^{6}+840\;x^{13}y^{12}\;\;\;+340\;x^{13}y^{18}+462\;x^{14}y^{6}+484\;x^{15}y^{6}+528\;x^{15}y^{8}+275\;x^{15}y^{9}+288\;x^{17}y^{8}\;\;\;+336\;x^{17}y^{12}+420\;x^{21}y^{14}+216\;x^{11}y^{8}+450\;x^{23}y^{14}\;\;\;+112\;x^{7})(1,1)=14376.$$ (6)

$$NFE(G_{1})=((d_{x}^{2}+d_{y}^{2})NEC(G_{1},x,y))(1,1)\;=(533\;x^{7}y^{22}+689\;x^{8}y^{25}+500\;x^{10}y^{20}+452\;x^{14}y^{16}+637\;x^{14}y^{21}+1040\;x^{16}y^{28}\;\;\;+900\;x^{18}y^{24}+724\;x^{18}y^{20}+1224\;x^{18}y^{30}+1556\;x^{20}y^{34}+1109\;x^{22}y^{25}\;\;\;+1152\;x^{24}y^{24}+1360\;x^{24}y^{28}+1800\;x^{30}y^{30}+2000\;x^{10}y^{30}+2554\;x^{11}y^{34}\;\;\;+1768\;x^{20}y^{22}+3552\;x^{20}y^{28}+1684\;x^{28}y^{30}+1850\;x^{21}y^{22}\;\;\;+1936\;x^{22}y^{22}+2120\;x^{22}y^{24}+949\;x^{18}y^{25})(1,1)=32089.$$ (7)

$$NRE(G_{1})=((d_{x}d_{y}(d_{x}+d_{y}))NEC(G_{1},x,y))(1,1)\;=(2\;x^{7}y^{16}(2233\;y^{6}+3300\;xy^{9}+3000\;x^{3}y^{4}+12000\;x^{3}y^{14}\;\;\;+16830\;x^{4}y^{18}+3360\;x^{7}+5145\;x^{7}y^{5}+9856\;x^{9}y^{12}+9072\;x^{11}y^{8}\;\;\;+6840\;x^{11}y^{4}+12960\;x^{11}y^{14}+18480\;x^{13}y^{6}+40320\;x^{13}y^{12}+24288\;x^{15}y^{8}\;\;\;+13824\;x^{17}y^{8}+17472\;x^{17}y^{12}+24360\;x^{21}y^{14}+27000\;x^{23}y^{14}+9675\;x^{11}y^{9}\;\;\;+12925\;x^{15}y^{9}+19866\;x^{14}y^{6}+21296\;x^{15}y^{6}\;\;\;+18360\;x^{13}y^{18})(1,1)=664924.$$ (8)

$$NSD_{\text{deg}}E(G_{1})=((d_{x}S_{y}+S_{x}d_{y})NEC(G_{1},x,y))(1,1)\;=(\frac{533}{154}\;x^{7}y^{22}+\frac{689}{200}\;x^{8}y^{25}+5/2\;x^{10}y^{20}+\frac{113}{56}\;x^{14}y^{16}+\frac{13}{6}\;x^{14}y^{21}\;\;\;+\frac{65}{28}\;x^{16}y^{28}+\frac{25}{12}\;x^{18}y^{24}+\frac{949}{450}\;x^{18}y^{25}+\frac{181}{90}\;x^{18}y^{20}+\frac{34}{15}\;x^{18}y^{30}\;\;\;+\frac{389}{170}\;x^{20}y^{34}+\frac{1109}{550}\;x^{22}y^{25}+2\;x^{24}y^{24}+\frac{85}{42}\;x^{24}y^{28}+\frac{421}{210}\;x^{28}y^{30}\;\;\;+2\;x^{30}y^{30}+\frac{20}{3}\;x^{10}y^{30}+\frac{1277}{187}\;x^{11}y^{34}+\frac{221}{55}\;x^{20}y^{22}+\frac{222}{35}\;x^{20}y^{28}\;\;\;+\frac{925}{231}\;x^{21}y^{22}+4\;x^{22}y^{22}+\frac{265}{66}\;x^{22}y^{24})(1,1)=72.5912.$$ (9)

$$NISIE(G_{1})=((S_{x}Jd_{x}d_{y})NEC(G_{1},x,y))(1,1)\;=(15\;x^{60}+\frac{420}{29}\;x^{58}+\frac{340}{27}\;x^{54}+\frac{168}{13}\;x^{52}+\frac{233}{4}\;x^{48}+\frac{550}{47}\;x^{47}+\frac{528}{23}\;x^{46}\;\;\;+\frac{748}{45}\;x^{45}+\frac{354}{11}\;x^{44}+\frac{656}{21}\;x^{42}+15\;x^{40}+\frac{180}{19}\;x^{38}+\frac{42}{5}\;x^{35}\;\;\;+\frac{200}{33}\;x^{33}+\frac{212}{15}\;x^{30}+\frac{154}{29}\;x^{29}+\frac{1374}{43}\;x^{43})(1,1)=318.2806.$$ (10)

$$NHE(G_{1})=((2S_{x}J)NEC(G_{1},x,y))(1,1)\;=(\frac{1}{30}\;x^{60}+\frac{1}{29}\;x^{58}+\frac{1}{27}\;x^{54}+\frac{1}{26}\;x^{52}+\frac{5}{24}\;x^{48}+\frac{2}{47}\;x^{47}+2/23\;x^{46}+\frac{4}{45}\;x^{45}\;\;\;+\frac{3}{22}\;x^{44}+\frac{6}{43}\;x^{43}+\frac{1}{7}\;x^{42}+\frac{1}{10}\;x^{40}+\frac{1}{19}\;x^{38}\;\;\;+\frac{2}{35}\;x^{35}+\frac{2}{33}\;x^{33}+\frac{2}{15}\;x^{30}+\frac{2}{29}\;x^{29})(1,1)=1.4614.$$ (11)

$$NAE(G_{1})=((S_{x}^{3}\Psi_{-2}Jd_{x}^{3}d_{y}^{3})NEC(G_{1},x,y))(1,1)\;=(\frac{91125000}{24389}\;x^{58}+3375\;x^{56}+\frac{4913000}{2197}\;x^{52}+\frac{37933056}{15625}\;x^{50}+\frac{109426872}{12167}\;x^{46}\;\;\;+\frac{1331000}{729}\;x^{45}+3456\;x^{44}+\frac{104627248}{79507}\;x^{43}+\frac{13194800}{3087}\;x^{42}+\frac{288347256}{68921}\;x^{41}\;\;\;+\frac{490214}{125}\;x^{40}+\frac{6750000}{6859}\;x^{38}+1000\;x^{36}+\frac{941192}{1331}\;x^{33}+\frac{8000000}{29791}\;x^{31}\;\;\;+\frac{300616}{343}\;x^{28}+\frac{3652264}{19683}\;x^{27})(1,1)=43768.2669.$$ (12)

◻

**Theorem 3.** *Let G_2_ be the graph of chloroquine. NEC-polynomial of G_2_ is given as follows:*

$$NEC(G_{2},x,y)=x^{7}y^{23}+2\;x^{12}y^{24}+x^{12}y^{35}+x^{15}y^{23}+x^{15}y^{25}+x^{16}y^{18}+x^{16}y^{23}\;\;\;+2\;x^{18}y^{20}+x^{18}y^{25}+x^{20}y^{20}+x^{20}y^{22}+x^{20}y^{28}+x^{20}y^{31}\;\;\;+x^{20}y^{34}+x^{22}y^{31}+2\;x^{22}y^{35}+2\;x^{24}y^{34}+x^{25}y^{28}+x^{28}y^{31}.$$ (13)

*Proof:* From Fig 2, it is easy to see that |V|=22 and |E|=23. The frequencies are given as |*E*_7,23_| = 1, |*E*_12,24_| = 2, |*E*_12,35_| = 1, |*E*_15,23_| = 1, |*E*_15,25_| = 1, |*E*_16,18_| = 1, |*E*_16,23_| = 1, |*E*_18,20_| = 2, |*E*_18,25_| = 1, |*E*_20,20_| = 1, |*E*_20,22_| = 1, |*E*_20,28_| = 1, |*E*_20,31_| = 1, |*E*_20,34_| = 1, |*E*_22,31_| = 1, |*E*_22,35_| = 2, |*E*_24,34_| = 2, |*E*_25,28_| = 1 and |*E*_28,31_| = 1. Then, we have


$$NEC(G_{2},x,y)=x^{7}y^{23}+2\;x^{12}y^{24}+x^{12}y^{35}+x^{15}y^{23}+x^{15}y^{25}+x^{16}y^{18}+x^{16}y^{23}\;\;\;+2\;x^{18}y^{20}+x^{18}y^{25}+x^{20}y^{20}+x^{20}y^{22}+x^{20}y^{28}+x^{20}y^{31}\;\;\;+x^{20}y^{34}+x^{22}y^{31}+2\;x^{22}y^{35}+2\;x^{24}y^{34}+x^{25}y^{28}+x^{28}y^{31}.$$

◻

**Theorem 4.** *Let G_2_ be the graph of chloroquine. Using Fig 2, the various topological indices of the G_2_ graph are as follows:*

1. NE_1_(G_2_)=1049
2. NE_2_(G_2_)=11825
3. NFE(G_2_)=26019
4. NRE(G_2_)=580864
5. NSD_deg_E (G_2_)=52.0127
6. NISIE(G_2_)=248.9108
7. NHE(G_2_)=1.0488
8. NAE (G_2_)=37842.6494

**Theorem 5.** *Let G_3_ be the graph of hydroxy-chloroquine. NEC-polynomial of G_3_ is given as follows:*

$$NEC(G_{3},x,y)=x^{7}y^{24}+x^{12}y^{24}+x^{13}y^{26}+x^{13}y^{38}+x^{16}y^{18}+2\;x^{16}y^{24}+x^{16}y^{28}\;\;\;+x^{18}y^{20}+x^{20}y^{22}+x^{20}y^{28}+x^{20}y^{34}+x^{22}y^{22}+x^{22}y^{24}+x^{22}y^{31}\;\;\;+x^{22}y^{34}+x^{24}y^{26}+3\;x^{24}y^{34}+2x^{24}y^{38}+x^{28}y^{31}+x^{31}y^{34}.$$ (14)

*Proof:* From Fig 3, it is easy to see that |V|=23 and |E|=24. The frequencies are given as |*E*_7,24_| = 1, |*E*_12,24_| = 1, |*E*_13,26_| = 1, |*E*_13,38_| = 1, |*E*_16,18_| = 1, |*E*_16,24_| = 2, |*E*_16,28_| = 1, |*E*_18,20_| = 1, |*E*_20,22_| = 1, |*E*_20,28_| = 1, |*E*_20,34_| = 1, |*E*_22,22_| = 1, |*E*_22,24_| = 1, |*E*_22,31_| = 1, |*E*_22,34_| = 1, |*E*_24,26_| = 1, |*E*_24,34_| = 3, |*E*_24,38_| = 2, |*E*_28,31_| = 1 and |*E*_31,34_| = 1. Then, we have


$$NEC(G_{3},x,y)=x^{7}y^{24}+x^{12}y^{24}+x^{13}y^{26}+x^{13}y^{38}+x^{16}y^{18}+2\;x^{16}y^{24}+x^{16}y^{28}\;\;\;+x^{18}y^{20}+x^{20}y^{22}+x^{20}y^{28}+x^{20}y^{34}+x^{22}y^{22}+x^{22}y^{24}+x^{22}y^{31}\;\;\;+x^{22}y^{34}+x^{24}y^{26}+3\;x^{24}y^{34}+2x^{24}y^{38}+x^{28}y^{31}+x^{31}y^{34}.$$

◻

**Theorem 6.** *Let G_3_ be the graph of hydroxy-chloroquine. Using Fig 3, the various topological indices of the G_3_ graph are as follows:*

1. NE_1_(G_3_)=1168
2. NE_2_(G_3_)=14092
3. NFE(G_3_)=30898
4. NRE(G_3_)=739548
5. NSD_deg_E (G_3_)=54.2184
6. NISIE(G_3_)=277.5706
7. NHE(G_3_)=1.0288
8. NAE (G_3_)=47990.9784

**Theorem 7.** *Let G_4_ be the graph of lopinavir. NEC-polynomial of G_4_ is given as follows:*

$$NEC(G_{4},x,y)=x^{9}y^{30}+x^{12}y^{37}+x^{13}y^{40}+x^{20}y^{30}+x^{20}y^{34}+2x^{28}y^{30}+2x^{28}y^{40}\;\;\;+x^{28}y^{46}+x^{30}y^{31}+x^{32}y^{34}+x^{32}y^{46}+x^{40}y^{43}+2\;x^{15}y^{46}\;\;\;+3\;x^{16}y^{49}+2\;x^{22}y^{31}+2\;x^{22}y^{37}+2\;x^{24}y^{34}+2\;x^{24}y^{40}+3\;x^{26}y^{28}\;\;\;+3\;x^{26}y^{37}+2\;x^{28}y^{28}+2\;x^{30}y^{30}+4\;x^{34}y^{34}+3\;x^{34}y^{49}\;\;\;+2\;x^{43}y^{46}+3\;x^{46}y^{49}.$$ (15)

*Proof:* From Fig 4, it is easy to see that |V|=46 and |E|=49. The frequencies are given as |*E*_9,30_| = 1, |*E*_12,37_| = 1, |*E*_13,40_| = 1, |*E*_20,30_| = 1, |*E*_20,34_| = 1, |*E*_28,30_| = 2, |*E*_28,40_| = 2, |*E*_28,46_| = 1, |*E*_30,31_| = 1,|*E*_32,34_| = 1, |*E*_32,46_| = 1, |*E*_40,43_| = 1, |*E*_15,46_| = 2, |*E*_16,49_| = 3, |*E*_22,31_| = 2, |*E*_22,37_| = 2, |*E*_24,34_| = 2, |*E*_24,40_| = 2, |*E*_26,28_| = 3, |*E*_26,37_| = 3, |*E*_28,28_| = 2, |*E*_30,30_| = 2, |*E*_34,34_| = 4, |*E*_34,49_| = 3, |*E*_43,46_| = 2, and |*E*_46,49_| = 3. Then, we have


$$NEC(G_{4},x,y)=x^{9}y^{30}+x^{12}y^{37}+x^{13}y^{40}+x^{20}y^{30}+x^{20}y^{34}+2x^{28}y^{30}+2x^{28}y^{40}\;\;\;+x^{28}y^{46}+x^{30}y^{31}+x^{32}y^{34}+x^{32}y^{46}+x^{40}y^{43}+2\;x^{15}y^{46}\;\;\;+3\;x^{16}y^{49}+2\;x^{22}y^{31}+2\;x^{22}y^{37}+2\;x^{24}y^{34}+2\;x^{24}y^{40}+3\;x^{26}y^{28}\;\;\;+3\;x^{26}y^{37}+2\;x^{28}y^{28}+2\;x^{30}y^{30}+4\;x^{34}y^{34}+3\;x^{34}y^{49}\;\;\;+2\;x^{43}y^{46}+3\;x^{46}y^{49}.$$

◻

**Theorem 8.** *Let G_4_ be the graph of lopinavir. Using Fig 4, the various topological indices of the G_4_ graph are as follows:*

1. NE_1_(G_4_)=3211
2. NE_2_(G_4_)=51986
3. NFE(G_4_)=114501
4. NRE(G_4_)=3699994
5. NSD_deg_E (G_4_)=112.9006
6. NISIE(G_4_)=759.3995
7. NHE(G_4_)=1.5496
8. NAE (G_4_)=235002.9451

**Theorem 9.** *Let G_5_ be the graph of remdesivir. NEC-polynomial of G_5_ is given as follows:*

$$NEC(G_{5},x,y)=x^{10}y^{42}+x^{13}y^{39}+x^{13}y^{53}+x^{13}y^{40}+x^{17}y^{52}+x^{20}y^{20}+x^{20}y^{34}\;\;\;+x^{20}y^{42}+x^{24}y^{34}+x^{24}y^{53}+x^{28}y^{30}+x^{28}y^{40}+x^{30}y^{31}+x^{30}y^{49}\;\;\;+x^{30}y^{43}+x^{31}y^{48}+x^{32}y^{34}+x^{32}y^{46}+x^{34}y^{34}+x^{34}y^{37}\;\;\;+x^{34}y^{52}+x^{37}y^{39}+x^{37}y^{40}+x^{39}y^{53}+x^{43}y^{46}+x^{43}y^{53}+x^{46}y^{48}\;\;\;+x^{48}y^{52}+2\;x^{12}y^{37}+2\;x^{17}y^{34}+2\;x^{22}y^{37}+2\;x^{22}y^{42}\;\;\;+2\;x^{26}y^{28}+2\;x^{26}y^{37}+2\;x^{28}y^{28}+2\;x^{34}y^{49}.$$ (16)

*Proof:* From Fig 5, it is easy to see that |V|=41 and |E|=44. The frequencies are given as |*E*_10,42_| = 1, |*E*_13,39_| = 1, |*E*_13,40_| = 1, |*E*_13,53_| = 1, |*E*_17,52_| = 1, |*E*_20,20_| = 1, |*E*_20,34_| = 1, |*E*_20,42_| = 1, |*E*_24,34_| = 1, |*E*_24,53_| = 1, |*E*_28,30_| = 1, |*E*_28,40_| = 1, |*E*_30,31_| = 1, |*E*_30,49_| = 1, |*E*_30,43_| = 1, |*E*_31,48_| = , |*E*_32,34_| = 1, |*E*_32,46_| = 1, |*E*_34,34_| = 1, |*E*_34,37_| = 1, |*E*_34,52_| = 1, |*E*_37,39_| = 1, |*E*_37,40_| = 1, |*E*_39,53_| = 1, |*E*_43,46_| = 1, |*E*_43,53_| = 1, |*E*_46,48_| = 1, |*E*_48,52_| = 1, |*E*_12,37_| = 2, |*E*_17,34_| = 2, |*E*_22,37_| = 2, |*E*_22,42_| = 2, |*E*_26,28_| = 2, |*E*_26,37_| = 2, |*E*_28,28_| = 2, and |*E*_34,49_| = 2. Then, we have


$$NEC(G_{5},x,y)=x^{10}y^{42}+x^{13}y^{39}+x^{13}y^{53}+x^{13}y^{40}+x^{17}y^{52}+x^{20}y^{20}+x^{20}y^{34}\;\;\;+x^{20}y^{42}+x^{24}y^{34}+x^{24}y^{53}+x^{28}y^{30}+x^{28}y^{40}+x^{30}y^{31}+x^{30}y^{49}\;\;\;+x^{30}y^{43}+x^{31}y^{48}+x^{32}y^{34}+x^{32}y^{46}+x^{34}y^{34}+x^{34}y^{37}\;\;\;+x^{34}y^{52}+x^{37}y^{39}+x^{37}y^{40}+x^{39}y^{53}+x^{43}y^{46}+x^{43}y^{53}+x^{46}y^{48}\;\;\;+x^{48}y^{52}+2\;x^{12}y^{37}+2\;x^{17}y^{34}+2\;x^{22}y^{37}+2\;x^{22}y^{42}\;\;\;+2\;x^{26}y^{28}+2\;x^{26}y^{37}+2\;x^{28}y^{28}+2\;x^{34}y^{49}.$$

◻

**Theorem 10.** *Let G_5_ be the graph of remdesivir. Using Fig 5, the various topological indices of the G_5_ graph are as follows:*

1. NE_1_(G_5_)=2952
2. NE_2_(G_5_)=48659
3. NFE(G_5_)=109810
4. NRE(G_5_)=3595128
5. NSD_deg_E (G_5_)=105.4888
6. NISIE(G_5_)=687.5585
7. NHE(G_5_)=1.3713
8. NAE (G_5_)=223477.2482

**Theorem 11.** *Let G_6_ be the graph of ritonavir. NEC-polynomial of G_6_ is given as follows:*

$$NEC(G_{6},x,y)=x^{12}y^{37}+x^{15}y^{46}+x^{16}y^{49}+x^{17}y^{52}+x^{28}y^{40}+x^{28}y^{46}+x^{34}y^{49}\;\;\;+x^{34}y^{55}+x^{37}y^{40}+x^{38}y^{39}+x^{38}y^{60}+x^{38}y^{61}+x^{39}y^{60}+x^{40}y^{43}\;\;\;+x^{43}y^{43}+x^{43}y^{46}+x^{46}y^{49}+x^{60}y^{64}+3\;x^{14}y^{43}+2\;x^{21}y^{64}+2\;x^{24}y^{37}\;\;\;+2\;x^{26}y^{37}+2\;x^{26}y^{43}+2\;x^{30}y^{32}+2\;x^{30}y^{43}+2\;x^{32}y^{32}+2\;x^{32}y^{46}\;\;\;+2\;x^{32}y^{52}+3\;x^{36}y^{38}+3\;x^{36}y^{52}+2\;x^{38}y^{38}+2\;x^{38}y^{55}\;\;\;+2\;x^{42}y^{43}+2\;x^{42}y^{61}.$$ (17)

*Proof:* From Fig 6, it is easy to see that |V|=50 and |E|=53. The frequencies are given as |*E*_12,37_| = 1, |*E*_15,46_| = 1, |*E*_16,49_| = 1, |*E*_17,52_| = 1, |*E*_28,40_| = 1, |*E*_28,46_| = 1, |*E*_34,49_| = 1, |*E*_34,55_| = 1, |*E*_37,40_| = 1, |*E*_38,39_| = 1, |*E*_38,60_| = 1, |*E*_38,61_| = 1, |*E*_39,60_| = 1, |*E*_40,43_| = 1, |*E*_43,43_| = 1, |*E*_43,46_| = 1, |*E*_46,49_| = 1, |*E*_60,64_| = 1, |*E*_14,43_| = 3, |*E*_21,64_| = 2, |*E*_24,37_| = 2, |*E*_26,37_| = 2, |*E*_26,43_| = 2, |*E*_30,32_| = 2, |*E*_30,43_| = 2, |*E*_32,32_| = 2, |*E*_32,46_| = 2, |*E*_32,52_| = 2, |*E*_36,38_| = 3, |*E*_36,52_| = 3, |*E*_38,38_| = 2, |*E*_38,55_| = 2, |*E*_42,43_| = 2 and |*E*_42,61_| = 2. Then, we have


$$NEC(G_{6},x,y)=x^{12}y^{37}+x^{15}y^{46}+x^{16}y^{49}+x^{17}y^{52}+x^{28}y^{40}+x^{28}y^{46}+x^{34}y^{49}\;\;\;+x^{34}y^{55}+x^{37}y^{40}+x^{38}y^{39}+x^{38}y^{60}+x^{38}y^{61}+x^{39}y^{60}+x^{40}y^{43}\;\;\;+x^{43}y^{43}+x^{43}y^{46}+x^{46}y^{49}+x^{60}y^{64}+3\;x^{14}y^{43}+2\;x^{21}y^{64}+2\;x^{24}y^{37}\;\;\;+2\;x^{26}y^{37}+2\;x^{26}y^{43}+2\;x^{30}y^{32}+2\;x^{30}y^{43}+2\;x^{32}y^{32}+2\;x^{32}y^{46}\;\;\;+2\;x^{32}y^{52}+3\;x^{36}y^{38}+3\;x^{36}y^{52}+2\;x^{38}y^{38}+2\;x^{38}y^{55}\;\;\;+2\;x^{42}y^{43}+2\;x^{42}y^{61}.$$

◻

**Theorem 12.** *Let G_6_ be the graph of ritonavir. Using Fig 6, the various topological indices of the G_6_ graph are as follows:*

1. NE_1_(G_6_)=4134
2. NE_2_(G_6_)=79061
3. NFE(G_6_)=175662
4. NRE(G_6_)=6636558
5. NSD_deg_E (G_6_)=123.0674
6. NISIE(G_6_)=973.6367
7. NHE(G_6_)=1.4070
8. NAE (G_6_)=410483.7478

**Theorem 13.** *Let G_7_ be the graph of thalidomide. NEC-polynomial of G_7_ is given as follows:*

$$NEC(G_{7},x,y)=3\;x^{6}y^{19}+x^{8}y^{23}+2\;x^{14}y^{23}+x^{14}y^{19}+x^{12}y^{14}+x^{12}y^{17}+2\;x^{17}y^{17}\;\;\;+3\;x^{17}y^{19}+2\;x^{19}y^{21}+2\;x^{16}y^{21}+2\;x^{16}y^{17}+x^{21}y^{21}.$$ (18)

*Proof:* From Fig 7, it is easy to see that |V|=19 and |E|=21. The frequencies are given as |*E*_6,19_| = 3, |*E*_8,23_| = 1, |*E*_14,23_| = 2, |*E*_14,19_| = 1, |*E*_12,14_| = 1, |*E*_12,17_| = 1, |*E*_17,17_| = 2, |*E*_17,19_| = 3, |*E*_19,21_| = 2, |*E*_16,21_| = 2, |*E*_16,17_| = 2 and |*E*_21,21_| = 1. Then, we have


$$NEC(G_{7},x,y)=3\;x^{6}y^{19}+x^{8}y^{23}+2\;x^{14}y^{23}+x^{14}y^{19}+x^{12}y^{14}+x^{12}y^{17}+2\;x^{17}y^{17}\;\;\;+3\;x^{17}y^{19}+2\;x^{19}y^{21}+2\;x^{16}y^{21}+2\;x^{16}y^{17}+x^{21}y^{21}.$$

◻

**Theorem 14.** *Let G_7_ be the graph of thalidomide. Using Fig 7, the various topological indices of the G_7_ graph are as follows:*

1. NE_1_(G_7_)=706
2. NE_2_(G_7_)= 5810
3. NFE(G_7_)=12640
4. NRE(G_7_)=204938
5. NSD_deg_E (G_7_)=48.6270
6. NISIE(G_7_)=167.5911
7. NHE(G_7_)=1.2803
8. NAE (G_7_)=14405.3630

**Theorem 15.** *Let G_8_ be the graph of theaflavin. NEC-polynomial of G_8_ is given as follows:*

$$NEC(G_{8},x,y)=x^{11}y^{33}+x^{11}y^{34}+x^{12}y^{37}+x^{13}y^{40}+x^{15}y^{44}+x^{18}y^{27}+x^{18}y^{31}\;\;\;+x^{20}y^{28}+x^{20}y^{32}+x^{21}y^{31}+x^{21}y^{33}+x^{22}y^{31}+x^{22}y^{37}+x^{24}y^{38}\;\;\;+x^{24}y^{40}+x^{26}y^{37}+x^{26}y^{41}+x^{28}y^{38}+x^{28}y^{46}+x^{34}y^{37}+x^{37}y^{38}\;\;\;+x^{37}y^{40}+x^{40}y^{41}+x^{41}y^{46}+2\;x^{10}y^{31}+x^{11}y^{32}+2\;x^{15}y^{46}+2\;x^{24}y^{34}\;\;\;+2\;x^{27}y^{28}+3\;x^{28}y^{31}+2\;x^{28}y^{40}+2\;x^{28}y^{44}+2\;x^{30}y^{46}\;\;\;+x^{31}y^{32}+x^{31}y^{33}+2\;x^{31}y^{34}.$$ (19)

*Proof:* From Fig 8, it is easy to see that |V|=41 and |E|=46. The frequencies are given as |*E*_11,33_| = 1, |*E*_11,34_| = 1, |*E*_12,37_| = 1, |*E*_13,40_| = 1, |*E*_15,44_| = 1, |*E*_18,27_| = 1, |*E*_18,31_| = 1, |*E*_20,28_| = 1, |*E*_20,32_| = 1, |*E*_21,31_| = 1, |*E*_21,33_| = 1, |*E*_22,31_| = 1, |*E*_22,37_| = 1, |*E*_24,38_| = 1, |*E*_24,40_| = 1, |*E*_26,37_| = 1, |*E*_26,41_| = 1, |*E*_28,38_| = 1, |*E*_28,46_| = 1, |*E*_34,37_| = 1, |*E*_37,38_| = 1, |*E*_37,40_| = 1, |*E*_40,41_| = 1, |*E*_41,46_| = 1, |*E*_10,31_| = 2, |*E*_11,32_| = 1, |*E*_15,46_| = 2, |*E*_24,34_| = 2, |*E*_27,28_| = 2, |*E*_28,31_| = 3, |*E*_28,40_| = 2, |*E*_28,44_| = 2, |*E*_30,46_| = 2, |*E*_31,32_| = 1, |*E*_31,33_| = 1 and |*E*_31,34_| = 2. Then, we have


$$NEC(G_{8},x,y)=x^{11}y^{33}+x^{11}y^{34}+x^{12}y^{37}+x^{13}y^{40}+x^{15}y^{44}+x^{18}y^{27}+x^{18}y^{31}\;\;\;+x^{20}y^{28}+x^{20}y^{32}+x^{21}y^{31}+x^{21}y^{33}+x^{22}y^{31}+x^{22}y^{37}+x^{24}y^{38}\;\;\;+x^{24}y^{40}+x^{26}y^{37}+x^{26}y^{41}+x^{28}y^{38}+x^{28}y^{46}+x^{34}y^{37}+x^{37}y^{38}\;\;\;+x^{37}y^{40}+x^{40}y^{41}+x^{41}y^{46}+2\;x^{10}y^{31}+x^{11}y^{32}+2\;x^{15}y^{46}+2\;x^{24}y^{34}\;\;\;+2\;x^{27}y^{28}+3\;x^{28}y^{31}+2\;x^{28}y^{40}+2\;x^{28}y^{44}+2\;x^{30}y^{46}\;\;\;+x^{31}y^{32}+x^{31}y^{33}+2\;x^{31}y^{34}.$$

◻

**Theorem 16.** *Let G_8_ be the graph of theaflavin. Using Fig 8, the various topological indices of the G_8_ graph are as follows:*

1. NE_1_(G_8_)=2788
2. NE_2_(G_8_)=41054
3. NFE(G_8_)=92400
4. NRE(G_8_)=2667738
5. NSD_deg_E (G_8_)=109.8168
6. NISIE(G_8_)=650.1624
7. NHE(G_8_)=1.5703
8. NAE (G_8_)=167520.7917

## 4 Computation of statistical parameters

Table 2 shows eight physical properties such as boiling point (BP), enthalpy of vaporisation (E), flash point (FP), molar refraction (MR), polar surface area (PSA), polarizability (P), molar volume (MV), and molecular weight (MW), of COVID-19 drugs taken from Chemspider and PubChem databases [37,38], while Table 3 represents the topological indices calculated values in each drug in previous section. The relation between topological indices and physical properties of COVID-19 drugs are found with the help of QSPR modeling.

**Table 2:** Physico-chemical properties of COVID-19 drugs [38].

Drugs	BP (mmHg)	E(kj/mol)	FP (°C)	MR(cm^3^)	PSA(Å^2^)	P(cm^3^)	MV(cm^3^)	MW (g/mol)
Arbidol	591.800	91.500	311.700	121.900	80.000	48.300	347.30	477.40
Chloroquine	460.600	72.100	232.300	97.400	28.200	38.600	287.90	319.90
Hydroxy-chloroquine	516.700	83.000	266.300	99.000	48.400	39.200	285.40	335.90
Lopinavir	924.200	140.800	512.700	179.200	120.000	71.000	540.50	628.80
Remdesivir	-	-	-	149.500	204.000	59.300	409.00	602.60
Ritonavir	947.000	144.400	526.600	198.900	202.000	78.900	581.70	720.90
Thalidomide	487.800	79.400	248.800	65.200	83.600	25.900	161.00	258.23
Theaflavin	1003.900	153.500	336.500	137.300	218.000	54.400	301.00	564.50

**Table 3:** Topological indicies values of drugs used for COVID-19 treatment.

Drugs	NE_1_	NE_2_	NFE	NRE	NSD_deg_E	NISIE	NHE	NAE
Arbidol	1355	14376	32089	664924	72.5912	318.2806	1.4614	43768.2669
Chloroquine	1049	11825	26019	580864	52.0127	248.9108	1.0488	37842.6494
Hydroxy-Chloroquine	1168	14092	30898	739548	54.2184	277.5706	1.0288	47990.9784
Lopinavir	3211	51986	114501	3699994	112.9006	759.3995	1.5496	235002.9451
Remdesivir	2952	48659	109810	3595128	105.4888	687.5585	1.3713	223477.2482
Ritonavir	4134	79061	175662	6636558	123.0674	973.6367	1.407	410483.7478
Thalidomide	706	5810	12640	204938	48.627	167.5911	1.2803	14405.363
Theaflavin	2788	41054	92400	2667738	109.8168	650.1624	1.5703	167520.7917

### 4.1 Linear regression models

The linear regression models for all the considered topological indices are given in this subsection. The correlation coefficients in linear regression model is presented in Table 4. Tables 5–12 show the statistical parameters used in the QSPR models of topological indices. We have used linear regression model

Y=A+B[TI], (20)

**Table 4:** Correlation coefficients in linear regression model.

	BP	E	FP	MR	PSA	P	MV	MW
NE_1_	0.926	0.921	0.934	0.961	0.828	0.961	0.878	0.968
NE_2_	0.875	0.871	0.932	0.950	0.799	0.950	**0.885**	0.944
NFE	0.878	0.874	0.930	0.948	0.806	0.949	0.881	0.945
NRE	0.825	0.822	0.920	0.930	0.771	0.931	0.879	0.916
NSD_deg_E	**0.977**	**0.971**	0.902	0.935	**0.873**	0.935	0.821	**0.978**
NISIE	0.923	0.918	**0.937**	**0.962**	0.821	**0.963**	0.883	0.967
NHE	0.799	0.797	0.633	0.606	0.702	0.606	0.466	0.717
NAE	0.829	0.825	0.924	0.934	0.770	0.934	0.883	0.919

Highest correlation values are shown in bold.

**Table 5:** The statistical parameters employed in the linear regression model of NE_1_.

	${\text{R}}^{2}$	SE	F	A	B	p	Indicator
BP	0.857	100.264	29.928	352.791	0.171	0.0027	Sign
E	0.848	15.082	27.815	58.230	0.025	0.0032	Sign
FP	0.872	48.019	34.195	167.758	0.087	0.0020	Sign
MR	0.926	14.088	62.981	56.712	0.035	0.0005	Sign
PSA	0.679	45.506	10.596	16.457	0.046	0.0225	Sign
P	0.927	5.564	63.476	22.468	0.014	0.0005	Sign
MV	0.777	77.781	17.431	149.562	0.101	0.0086	Sign
MW	0.933	49.274	70.157	207.541	0.129	0.0003	Sign

**Table 6:** The statistical parameters employed in the linear regression model of NE_2_.

	${\text{R}}^{2}$	SE	F	A	B	p	Indicator
BP	0.766	128.221	16.358	461.302	0.008	0.0098	Sign
E	0.758	18.992	15.693	73.949	0.001	0.0107	Sign
FP	0.869	48.579	33.296	216.346	0.004	0.0021	Sign
MR	0.903	16.161	46.657	76.630	0.002	0.0010	Sign
PSA	0.631	48.791	8.566	44.467	0.002	0.0327	Sign
P	0.904	6.376	47.152	30.361	0.001	0.0010	Sign
MV	0.787	75.978	18.509	204.494	0.005	0.0076	Sign
MW	0.884	64.937	38.274	283.776	0.006	0.0016	Sign

**Table 7:** The statistical parameters employed in the linear regression model of NFE.

	${\text{R}}^{2}$	SE	F	A	B	p	Indicator
BP	0.771	126.816	16.834	460.990	0.004	0.0093	Sign
E	0.764	18.789	16.143	73.901	0.001	0.0101	Sign
FP	0.864	49.561	31.793	217.017	0.002	0.0024	Sign
MR	0.901	16.320	45.659	76.790	0.001	0.0010	Sign
PSA	0.641	48.187	8.909	44.125	0.001	0.0306	Sign
P	0.902	6.439	46.135	30.425	0.0002	0.0010	Sign
MV	0.781	77.072	17.846	205.407	0.002	0.0082	Sign
MW	0.886	64.443	38.939	283.974	0.003	0.0015	Sign

**Table 8:** The statistical parameters employed in the linear regression model of NRE.

	${\text{R}}^{2}$	SE	F	A	B	p	Indicator
BP	0.681	149.580	10.694	520.403	8.484E-5	0.0221	Sign
E	0.675	22.023	10.390	82.515	1.231E-5	0.0233	Sign
FP	0.846	52.729	27.505	243.722	4.797E-5	0.0033	Sign
MR	0.864	19.179	31.681	87.770	1.872E-5	0.0024	Sign
PSA	0.589	51.527	7.164	59.530	2.392E-5	0.0439	Sign
P	0.865	7.568	32.015	34.777	7.428E-6	0.0023	Sign
MV	0.774	78.312	17.128	235.801	5.622E-5	0.0090	Sign
MW	0.832	78.362	24.717	325.550	6.758E-5	0.0042	Sign

**Table 9:** The statistical parameters employed in the linear regression model of NSD_deg_E.

	${\text{R}}^{2}$	SE	F	A	B	p	Indicator
BP	0.954	56.753	104.016	106.844	7.299	0.0001	Sign
E	0.944	9.161	83.948	22.568	1.058	0.0002	Sign
FP	0.813	58.136	21.740	67.920	3.418	0.0055	Sign
MR	0.878	18.168	35.876	16.040	1.372	0.0018	Sign
PSA	0.760	39.408	15.796	-50.284	1.975	0.0105	Sign
P	0.878	7.198	35.916	6.350	0.544	0.0018	Sign
MV	0.677	93.641	10.476	44.838	3.822	0.0230	Sign
MW	0.953	41.503	100.936	41.637	5.258	0.0001	Sign

**Table 10:** The statistical parameters employed in the linear regression model of NISIE.

	${\text{R}}^{2}$	SE	F	A	B	p	Indicator
BP	0.852	102.113	28.675	352.831	0.725	0.0030	Sign
E	0.842	15.346	26.696	58.239	0.105	0.0035	Sign
FP	0.878	47.045	35.835	166.686	0.373	0.0018	Sign
MR	0.929	13.852	65.318	56.401	0.148	0.0004	Sign
PSA	0.671	46.101	10.196	16.765	0.195	0.0241	Sign
P	0.929	5.470	65.851	22.345	0.059	0.0004	Sign
MV	0.784	76.649	18.098	148.071	0.432	0.0080	Sign
MW	0.932	49.738	68.762	206.923	0.547	0.0004	Sign

**Table 11:** The statistical parameters employed in the linear regression model of NHE.

	${\text{R}}^{2}$	SE	F	A	B	p	Indicator
BP	0.639	159.231	8.849	-447.691	863.007	0.0309	Sign
E	0.635	23.358	8.681	-58.175	125.390	0.0320	Sign
FP	0.401	104.066	3.345	-115.175	346.785	0.1269	Insign
MR	0.366	41.353	2.890	-42.589	128.076	0.1499	Insign
PSA	0.564	53.039	6.480	-216.997	246.002	0.0515	Insign
P	0.366	16.393	2.890	-16.890	50.772	0.1498	Insign
MV	0.214	146.039	1.363	-56.919	310.632	0.2956	Insign
MW	0.531	130.771	5.671	-285.306	567.372	0.0630	Sign

**Table 12:** The statistical parameters employed in the linear regression model of NAE.

	${\text{R}}^{2}$	SE	F	A	B	p	Indicator
BP	0.688	148.136	11.001	515.933	0.001	0.0210	Sign
E	0.681	21.820	10.678	81.868	0.0002	0.0222	Sign
FP	0.854	51.386	29.227	241.187	0.001	0.0029	Sign
MR	0.870	18.745	33.398	86.284	0.0003	0.0021	Sign
PSA	0.588	51.593	7.133	58.555	0.0003	0.0443	Sign
P	0.871	7.396	33.756	34.401	0.0001	0.0021	Sign
MV	0.780	77.200	17.770	232.885	0.001	0.0083	Sign
MW	0.837	77.148	25.659	322.196	0.001	0.0038	Sign

where, *Y* is the physical property of drug, *B* is the regression coefficient, *A* is the constant and *TI* is the topological index. *R* shows the correlation coefficient and *p* is the *p*-value. *SE* and *F* demonstrate the standard error of the estimates and the Fisher’s statistic [39], respectively. Constant *A* and regression coefficient *B* is calculated from SPSS software for eight physical properties and eight based topological indices of molecular structure of eight drugs. The relationship between the topological index and the physico-chemical properties of the drugs and the correlation coefficients associated with them are shown in Fig 1.

Using Eq (20), followings are the linear regression model for the defined based topological indices:

1. Regression models for first neighboorhood eccentricity index NE_1_:

$$\text{Boiling point}=352.791+0.171[NE_{1}].$$ (21)

$$\text{Enthalpy of vaporization}=58.230+0.025[NE_{1}].$$ (22)

$$\text{Flash point}=167.758+0.087[NE_{1}].$$ (23)

$$\text{Molar refractivity}=56.712+0.035[NE_{1}].$$ (24)

$$\text{Polar Surface Area}=16.457+0.046[NE_{1}].$$ (25)

$$\text{Polarizability}=22.468+0.014[NE_{1}].$$ (26)

$$\text{Molar volume}=149.562+0.101[NE_{1}].$$ (27)

$$\text{Molar weight}=207.541+0.129[NE_{1}].$$ (28)

2. Regression models for second neighboorhood eccentricity index NE_2_:

$$\text{Boiling point}=461.302+0.008[NE_{2}].$$ (29)

$$\text{Enthalpy of vaporization}=73.949+0.001[NE_{2}].$$ (30)

$$\text{Flash point}=216.346+0.004[NE_{2}].$$ (31)

$$\text{Molar refractivity}=76.630+0.002[NE_{2}].$$ (32)

$$\text{Polar Surface Area}=44.467+0.002[NE_{2}].$$ (33)

$$\text{Polarizability}=30.361+0.001[NE_{2}].$$ (34)

$$\text{Molar volume}=204.494+0.005[NE_{2}].$$ (35)

$$\text{Molar weight}=283.776+0.006[NE_{2}].$$ (36)

3. Regression models for forgotten neighboorhood eccentricity index NFE:

Boiling point=460.990+0.004[NFE]. (37)

Enthalpy of vaporization=73.901+0.001[NFE]. (38)

Flash point=217.017+0.002[NFE]. (39)

Molar refractivity=76.790+0.001[NFE]. (40)

Polar Surface Area=44.125+0.001[NFE]. (41)

Polarizability=30.425+0.0002[NFE]. (42)

Molar volume=205.407+0.002[NFE]. (43)

Molar weight=283.974+0.003[NFE]. (44)

4. Regression models for reformulate neighboorhood eccentricity index NRE:

Boiling point=520.403+8.484E-5[NRE]. (45)

Enthalpy of vaporization=82.515+1.231E-5[NRE]. (46)

Flash point=243.722+4.797E-5[NRE]. (47)

Molar refractivity=87.770+1.872E-5[NRE]. (48)

Polar Surface Area=59.530+2.392E-5[NRE]. (49)

Polarizability=34.777+7.428E-6[NRE]. (50)

Molar volume=235.801+5.622E-5[NRE]. (51)

Molar weight=325.550+6.758E-5[NRE]. (52)

5. Regression models for symmetric degree division neighboorhood eccentricity index NSD_deg_E :

$$\text{Boiling point}=106.844+7.299[NSD_{\text{deg}}E].$$ (53)

$$\text{Enthalpy of vaporization}=22.568+1.058[NSD_{\text{deg}}E].$$ (54)

$$\text{Flash point}=67.920+3.418[NSD_{\text{deg}}E].$$ (55)

$$\text{Molar refractivity}=16.040+1.372[NSD_{\text{deg}}E].$$ (56)

$$\text{Polar Surface Area}=-50.284+1.975[NSD_{\text{deg}}E].$$ (57)

$$\text{Polarizability}=6.350+0.544[NSD_{\text{deg}}E].$$ (58)

$$\text{Molar volume}=44.838+3.822[NSD_{\text{deg}}E].$$ (59)

$$\text{Molar weight}=41.637+5.258[NSD_{\text{deg}}E].$$ (60)

**Fig 9:**
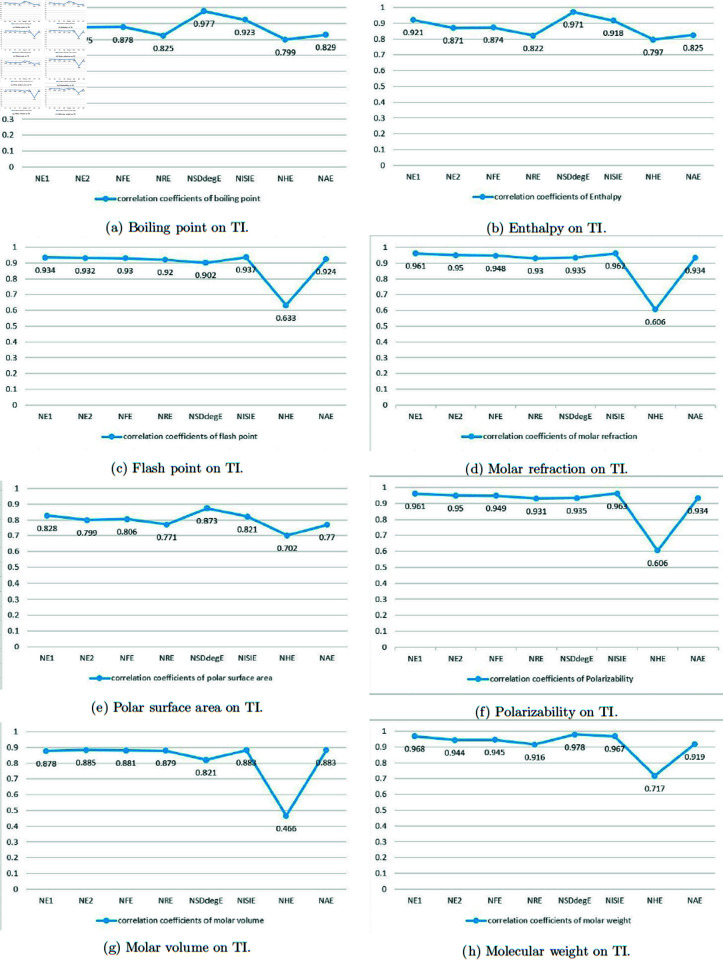
Correlation of physicochemical properties on TIs.

6. Regression models for inverse sum indegree neighborhood eccentricity index NISIE:

Boiling point=352.831+0.725[NISIE]. (61)

Enthalpy of vaporization=58.239+0.105[NISIE]. (62)

Flash point=166.686+0.373[NISIE]. (63)

Molar refractivity=56.401+0.148[NISIE]. (64)

Polar Surface Area=16.765+0.195[NISIE]. (65)

Polarizability=22.345+0.059[NISIE]. (66)

Molar volume=148.071+0.432[NISIE]. (67)

Molar weight=206.923+0.547[NISIE]. (68)

7. Regression models for harmonic neighborhood eccentricity index NHE:

Boiling point=−447.691+863.007[NHE]. (69)

Enthalpy of vaporization=−58.175+125.390[NHE]. (70)

Flash point=−115.175+346.785[NHE]. (71)

Molar refractivity=−42.589+128.076[NHE]. (72)

Polar Surface Area=−216.997+246.002[NHE]. (73)

Polarizability=−16.890+50.772[NHE]. (74)

Molar volume=−56.919+310.632[NHE]. (75)

Molar weight=−285.306+567.372[NHE]. (76)

8. Regression models for augmented neighborhood eccentricity index NAE:

Boiling point=515.933+0.001[NAE]. (77)

Enthalpy of vaporization=81.868+0.0002[NAE]. (78)

Flash point=241.187+0.001[NAE]. (79)

Molar refractivity=86.284+0.0003[NAE]. (80)

Polar Surface Area=58.555+0.0003[NAE]. (81)

Polarizability=34.401+0.0001[NAE]. (82)

Molar volume=232.885+0.001[NAE]. (83)

Molar weight=322.196+0.001[NAE]. (84)

### 4.2 Cubic regression model

In this part, the cubic regression model is tested. We have used the following cubic regression model

$$Y=A+B[TI]+C[TI{]}^{2}+D[TI{]}^{3},$$ (85)

where, *Y* is the physical property, *A* is the regression model constant, *B*, *C* and *D* are the coefficients for the individual descriptor. The correlation coefficients in cubic regression model is presented in Table 13. We determine the best possible cubic regression model for predicting physicochemical properties of COVID-19 drugs in Table 14.

**Table 13:** Correlation coefficients in cubic regression model.

	BP	E	FP	MR	PSA	P	MV	MW
NE_1_	**0.980**	**0.976**	0.938	0.976	0.842	0.976	0.906	**0.981**
NE_2_	0.966	0.962	0.949	0.966	0.814	0.966	0.891	0.966
NFE	0.969	0.964	0.945	0.966	0.820	0.966	0.889	0.968
NRE	0.962	0.958	0.963	0.958	0.819	0.958	0.888	0.956
NSD_deg_E	0.978	0.972	0.919	0.938	**0.876**	0.938	0.833	0.977
NISIE	0.978	0.974	0.942	**0.977**	0.836	**0.977**	**0.907**	**0.981**
NHE	0.838	0.834	0.634	0.615	0.751	0.615	0.466	0.734
NAE	0.962	0.957	**0.964**	0.959	0.818	0.959	0.891	0.957

Highest correlation values are shown in bold.

**Table 14:** Statistical parameters of best predictions in the cubic regression model.

	${\text{R}}^{2}$	SE	F	A	B	C	D	p	Indicator
BP-NE_1_	0.960	68.219	24.150	462.737	-0.140	0.0002	-3.650E-8	0.0132	Sign
E-NE_1_	0.953	10.802	20.325	81.066	-0.031	3.562E-5	-5.909E-9	0.0169	Sign
FP-NAE	0.929	46.183	13.124	290.642	-0.001	1.619E-8	-2.743E-14	0.0312	Sign
MR-NISIE	0.952	12.891	26.510	-21.143	0.682	-0.001	5.423E-7	0.0042	Sign
PSA-NSD_deg_E	0.768	43.294	6.615	44.688	-0.590	0.015	0.000	0.0538	Insign
P-NISIE	0.954	5.701	20.742	-7.565	0.262	-0.0003	1.983E-7	0.0165	Sign
MV-NISIE	0.824	89.344	4.667	-98.771	2.240	-0.004	2.105E-6	0.1189	Insign
MW-NE_1_	0.963	47.411	26.060	-71.570	0.556	-0.0001	2.011E-8	0.0119	Sign
MW-NISIE	0.962	48.281	25.093	-72.220	2.353	-0.003	1.520E-6	0.0125	Sign

From Table 14, NE_1_ for BP, E and MW; NAE for FP; NISIE for MR, P, MV and MW; NSD_deg_E for PSA are the best estimator indices in cubic regression model.

$$\text{Boiling Point}=462.737-0.140[NE_{1}]+0.0002[NE_{1}{]}^{2}-3.650E\text{-}8[NE_{1}{]}^{3}.$$ (86)

$$\text{Enthalpy of vaporization}=81.066-0.031[NE_{1}]+3.562E\text{-}5[NE_{1}{]}^{2}-5.909E\text{-}9[NE_{1}{]}^{3}.$$ (87)

$$\text{Flash point}=290.642-0.001[NAE]+1.619E\text{-}8[NAE{]}^{2}-2.743E\text{-}14[NAE{]}^{3}.$$ (88)

$$\text{Molar refractivity}=-21.143+0.682[NISIE]-0.001[NISIE{]}^{2}+5.423E\text{-}7[NISIE{]}^{3}.$$ (89)

$$\text{Polar Surface Area}=44.688-0.590[NSD_{\text{deg}}E]+0.015[NSD_{\text{deg}}E{]}^{2}+0.000[NSD_{\text{deg}}E{]}^{3}.$$ (90)

$$\text{Polarizability}=-7.565+0.262[NISIE]-0.0003[NISIE{]}^{2}+1.983E\text{-}7[NISIE{]}^{3}.$$ (91)

$$\text{Molar volume}=-98.771+2.240[NISIE]-0.004[NISIE{]}^{2}+2.105E\text{-}6[NISIE{]}^{3}.$$ (92)

$$\text{Molar weight}=-71.570+0.556[NE_{1}]-0.0001[NE_{1}{]}^{2}+2.011E\text{-}8[NE_{1}{]}^{3}.$$ (93)

$$\text{Molar weight}=-72.220+2.353[NISIE]-0.003[NISIE{]}^{2}+1.520E\text{-}6[NISIE{]}^{3}.$$ (94)

## 5 Comparison and discussions

The molecular topology of the COVID-19 drugs is modeled by means of several topological indices, and a QSPR analysis is carried out to investigate the predictive ability of the topological indices under consideration. We have correlated eight physical properties of drugs used to treat COVID-19 with neighborhood eccentricity based topological indices. This section presents the comparison for known values and calculated values from regression models. Tables 15–22 show the comparison of each physical property for linear regression models, while Tables 23 and 24 give the comparison of actual and calculated values for drugs from cubic regression models for the best predictors.

**Table 15:** Comparison of actual and computed values for boiling point from linear regression model.

Drugs	BP	NE_1_	NE_2_	NFE	NRE	NSD_deg_E	NISIE	NHE	NAE
Arbidol	591.8	584.496	576.31	589.346	576.815	636.687	583.584	813.507	559.701
Chloroquine	460.6	532.17	555.902	565.066	569.683	486.484	533.291	457.430	553.775
Hydroxy-Chloroquine	516.7	552.519	574.038	584.582	583.146	502.584	554.069	440.170	563.923
Lopinavir	924.2	901.872	877.19	918.994	834.310	930.905	903.395	889.624	750.935
Remdesivir		857.583	850.574	900.23	825.413	876.806	851.310	735.750	739.410
Ritonavir	947	1059.705	1093.79	1163.638	1083.448	1005.112	1058.717	766.559	926.416
Thalidomide	487.8	473.517	507.782	511.55	537.789	461.772	474.334	657.216	530.338
Theaflavin	1003.9	829.539	789.734	830.59	746.733	908.396	824.198	907.488	683.453

**Table 16:** Comparison of actual and computed values for enthalpy of vaporization from linear regression model.

Drugs	E	NE_1_	NE_2_	NFE	NRE	NSD_deg_E	NISIE	NHE	NAE
Arbidol	91.5	92.105	88.325	105.99	90.700	99.369	91.658	125.069	90.621
Chloroquine	72.1	84.455	85.774	99.92	89.665	77.597	84.374	73.334	89.436
Hydroxy-Chloroquine	83	87.43	88.041	104.799	91.618	79.931	87.383	70.826	91.466
Lopinavir	140.8	138.505	125.935	188.402	128.061	142.016	137.975	136.129	128.868
Remdesivir		132.03	122.608	183.711	126.771	134.175	130.432	113.772	126.563
Ritonavir	144.4	161.58	153.01	249.563	164.211	152.773	160.470	118.248	163.964
Thalidomide	79.4	75.88	79.759	86.541	85.037	74.015	75.836	102.361	84.749
Theaflavin	153.5	127.93	115.003	166.301	115.354	138.754	126.506	138.724	115.372

**Table 17:** Comparison of actual and computed values for flash point from linear regression model.

Drugs	FP	NE_1_	NE_2_	NFE	NRE	NSD_deg_E	NISIE	NHE	NAE
Arbidol	311.7	285.643	273.85	281.195	275.618	316.036	285.404	391.62	284.955
Chloroquine	232.3	259.021	263.646	269.055	271.586	245.699	259.529	248.53	279.029
Hydroxy-Chloroquine	266.3	269.374	272.714	278.813	279.198	253.238	270.219	241.6	289.177
Lopinavir	512.7	447.115	424.29	446.019	421.210	453.814	449.942	422.2	476.189
Remdesivir		424.582	410.982	436.637	416.180	428.480	423.145	360.37	464.664
Ritonavir	526.6	527.416	532.59	568.341	562.077	488.564	529.852	372.75	651.670
Thalidomide	248.8	229.18	239.586	242.297	253.552	234.127	229.197	328.81	255.592
Theaflavin	336.5	410.314	380.562	401.817	371.693	443.273	409.196	429.38	408.707

**Table 18:** Comparison of actual and computed values for molar refractivity from linear regression model.

Drugs	MR	NE_1_	NE_2_	NFE	NRE	NSD_deg_E	NISIE	NHE	NAE
Arbidol	121.9	104.137	105.382	108.879	100.217	115.635	103.506	391.616	99.414
Chloroquine	97.4	93.427	100.28	102.809	98.643	87.401	93.239	248.533	97.636
Hydroxy-Chloroquine	99	97.592	104.814	107.688	101.614	90.427	97.481	241.597	100.681
Lopinavir	179.2	169.097	180.602	191.291	157.033	170.939	168.792	422.203	156.784
Remdesivir	149.5	160.032	173.948	186.6	155.070	160.770	158.159	360.371	153.327
Ritonavir	198.9	201.402	234.752	252.452	212.006	184.888	200.499	372.751	209.429
Thalidomide	65.2	81.422	88.25	89.43	91.606	82.756	81.204	328.813	90.605
Theaflavin	137.3	154.292	158.738	169.19	137.710	166.708	152.625	429.381	136.540

**Table 19:** Comparison of actual and computed values for polar surface area from linear regression model.

Drugs	PSA	NE_1_	NE_2_	NFE	NRE	NSD_deg_E	NISIE	NHE	NAE
Arbidol	80	78.787	73.219	76.214	75.434	93.083	78.829	142.510	71.685
Chloroquine	28.2	64.711	68.117	70.144	73.424	52.441	65.302	41.009	69.907
Hydroxy-Chloroquine	48.4	70.185	72.651	75.023	77.219	56.797	70.891	36.089	72.952
Lopinavir	120	164.163	148.439	158.626	148.033	172.694	164.847	164.207	129.055
Remdesivir	204	152.249	141.785	153.935	145.525	158.056	150.838	120.345	125.598
Ritonavir	202	206.621	202.589	219.787	218.276	192.774	206.624	129.127	181.700
Thalidomide	83.6	48.933	56.087	56.765	64.432	45.754	49.445	97.959	62.876
Theaflavin	218	144.705	126.575	136.525	123.342	166.604	143.546	169.299	108.811

**Table 20:** Comparison of actual and computed values for polarizability from linear regression model.

Drugs	P	NE_1_	NE_2_	NFE	NRE	NSD_deg_E	NISIE	NHE	NAE
Arbidol	48.3	41.438	44.737	36.842	39.716	45.839	41.123	57.308	38.777
Chloroquine	38.6	37.154	42.186	35.6288	39.091	34.644	37.030	36.359	38.185
Hydroxy-Chloroquine	39.2	38.82	44.453	36.604	40.270	35.844	38.721	35.344	39.2
Lopinavir	71	67.422	82.347	53.325	62.260	67.767	67.149	61.786	57.901
Remdesivir	59.3	63.796	79.02	52.387	61.481	63.735	62.910	52.733	56.748
Ritonavir	78.9	80.344	109.422	65.557	84.073	73.298	79.789	54.546	75.449
Thalidomide	25.9	32.352	36.171	32.953	36.299	32.803	32.232	48.113	35.841
Theaflavin	54.4	61.5	71.415	48.905	54.592	66.090	60.704	62.837	51.153

**Table 21:** Comparison of actual and computed values for molar volume from linear regression model.

Drugs	MV	NE_1_	NE_2_	NFE	NRE	NSD_deg_E	NISIE	NHE	NAE
Arbidol	347.3	286.417	276.374	269.585	273.183	322.281	285.568	397.038	276.653
Chloroquine	287.9	255.511	263.619	257.445	268.457	243.630	255.6	268.871	270.727
Hydroxy-Chloroquine	285.4	267.53	274.954	267.203	277.378	252.060	267.981	262.659	280.875
Lopinavir	540.5	473.873	464.424	434.409	443.814	476.344	476.131	424.436	467.887
Remdesivir	409	447.714	447.789	425.027	437.919	448.016	445.096	369.050	456.362
Ritonavir	581.7	567.096	599.799	556.731	608.908	515.201	568.682	380.140	643.368
Thalidomide	161	220.868	233.544	230.687	247.322	230.69	220.47	340.783	247.29
Theaflavin	301	431.15	409.764	390.207	385.781	464.557	428.941	430.866	400.405

**Table 22:** Comparison of actual and computed values for molar weight from linear regression model.

Drugs	MW	NE_1_	NE_2_	NFE	NRE	NSD_deg_E	NISIE	NHE	NAE
Arbidol	477.4	382.336	370.032	380.241	370.485	423.321	381.022	543.851	365.964
Chloroquine	319.9	342.862	354.726	362.031	364.804	315.119	343.077	309.753	360.038
Hydroxy-Chloroquine	335.9	358.213	368.328	376.668	375.528	326.717	358.754	298.406	370.186
Lopinavir	628.8	621.76	595.692	627.477	575.595	635.268	622.314	593.893	557.198
Remdesivir	602.6	588.349	575.73	613.404	568.508	596.297	583.017	492.731	545.673
Ritonavir	720.9	740.827	758.142	810.96	774.048	688.725	739.502	512.986	732.679
Thalidomide	258.23	298.615	318.636	321.894	339.399	297.317	298.595	441.100	336.601
Theaflavin	564.5	567.193	530.1	561.174	505.835	619.053	562.561	605.638	489.716

**Table 23:** Comparison of actual and computed values for drugs from cubic regression models for best predictors.

Drugs	BP	NE_1_	E	NE_1_	FP	NAE	MR	NISIE
Arbidol	591.8	549.44	91.5	89.76	311.7	275.59	121.9	112.11
Chloroquine	460.6	493.82	72.1	80.92	232.3	274.5	97.4	95.02
Hydroxy-Chloroquine	516.7	513.9	83	84.04	266.3	276.91	99	102.71
Lopinavir	924.2	866.89	140.8	153.16	512.7	593.76	179.2	157.57
Remdesivir		853.37		147.95		569.58	149.5	151.3
Ritonavir	947	723.25	144.4	144.19	526.6	710.92	198.9	195.44
Thalidomide	487.8	450.74	79.4	74.85	248.8	279.51	65.2	67.62
Theaflavin	1003.9	836.02	153.5	143.46	336.5	448.51	137.3	148.6

**Table 24:** Comparison of actual and computed values for drugs from cubic regression models for best predictors.

Drugs	PSA	NSD_deg_E	P	NISIE	MV	NISIE	MW	NE_1_	NISIE
Arbidol	80	80.9	48.3	51.83	347.3	276.84	477.4	548.24	421.8
Chloroquine	28.2	54.58	38.6	42.12	287.9	243.43	319.9	424.85	351.04
Hydroxy-Chloroquine	48.4	56.79	39.2	46.29	285.4	259.82	335.9	473.46	382.27
Lopinavir	120	169.27	71	105.23	540.5	217.39	628.8	1348.48	650.25
Remdesivir	204	149.37	59.3	95.21	409	234.61	602.6	1215.63	621.45
Ritonavir	202	199.26	78.9	146.16	581.7	233.17	720.9	1938.71	777.77
Thalidomide	83.6	51.47	25.9	28.85	161	174.19	258.23	278.2	245.02
Theaflavin	218	160.79	54.4	90.46	301	245.27	564.5	1137.07	607.22

The linear QSPR analysis (see Table 4) illustrates that NE_2_ index is the best predictor for MV; NSD_deg_E index has high correlated value for BP, E, PSA and MW; and NISIE index gives high correlation coefficients for FP, MR and P. On the other hand, in cubic regression model (see Table 13), NE_1_ index is the best predictor for BP, E and MW; NSD_deg_E index has high correlated for PSA; NISIE index is the best predictor for MR, P, MV and MW; and NAE index gives best correlation values for FP.

## 6 Conclusion

In this work, the eight topological indices for eight drugs utilized in the treatment of COVID-19 disease, namely arbidol, chloroquine, hydroxy-chloroquine, lopinavir, remdesivir, ritonavir, thalidomide, and theaflavin, which are shown in Figs 1–8 have been evaluated. The correlation between these indices and eight physico-chemical properties, namely boiling point, enthalpy of vaporization, flash point, molar refraction, polar surface area, polarizability, molar volume, and molecular weight has been analyzed, and correlation coefficients for linear and cubic models have been presented in Tables 4 and 13. The results show that certain topological indices had strong correlations with specific physico-chemical properties. Additionally, SPSS software to fit linear and cubic regression models to predict the physico-chemical properties has been used. All the statistical parameters for linear model have been presented in Tables 5–12.

These analyses represent that the *p*-value in each case except for FP, MR, PSA, P and MV with harmonic neighborhood eccentricity index is less than 0.05, which indicates the significance of the results. The best predictor indices for each property in the cubic model have been identified (see Table 14). It is observed from Tables 15–24 that the computed values of the properties are found to be close to the actual values, further validating the predictive power of these indices. It is found that the second neighborhood eccentricity index is the best predictor for molar volume in linear regression models. Boiling point, enthalpy, polar surface area and molecular weight properties are best predicted by the symmetric degree division neighborhood eccentricity index in linear regression models. Flash point, molar refraction and polarizability properties are best predicted by inverse sum indegree neighborhood eccentricity in linear regression models.

In cubic regression equations, boiling point, enthalpy vaporization and molecular weight are best predicted by the first neighborhood eccentricity index. The symmetric degree division neighborhood eccentricity index is suitable for polar surface area. The augmented neighborhood eccentricity index is the best predictor for flash point and the other properties such as molar refraction, polarization, molar volume and molecular weight are best predicted by the inverse sum indegree neighborhood eccentricity index. The QSPR analysis of the paper could be helpful in the drug’s development against corona viruses. To our best knowledge, this is the first investigation on various COVID-19 drugs chosen using neighborhood eccentricity-based topological descriptors.
